# Large-scale sill emplacement in Brazil as a trigger for the end-Triassic crisis

**DOI:** 10.1038/s41598-017-18629-8

**Published:** 2018-01-09

**Authors:** Thea H. Heimdal, Henrik. H. Svensen, Jahandar Ramezani, Karthik Iyer, Egberto Pereira, René Rodrigues, Morgan T. Jones, Sara Callegaro

**Affiliations:** 1 Centre for Earth Evolution and Dynamics (CEED), University of Oslo, PO Box 1047, Blindern, NO-0316 Oslo Norway; 2 Department of Earth, Atmospheric and Planetary Sciences, Massachusetts Institute of Technology, Cambridge, MA 02139 USA; 3 GeoModelling Solutions GmbH, Zürich, Switzerland; 4 GEOMAR, Helmholtz Institute for Ocean Research, Kiel, Germany; 5 Department of Stratigraphy and Paleontology, Rio de Janeiro State University, Rio de Janeiro, Brazil

## Abstract

The end-Triassic is characterized by one of the largest mass extinctions in the Phanerozoic, coinciding with major carbon cycle perturbations and global warming. It has been suggested that the environmental crisis is linked to widespread sill intrusions during magmatism associated with the Central Atlantic Magmatic Province (CAMP). Sub-volcanic sills are abundant in two of the largest onshore sedimentary basins in Brazil, the Amazonas and Solimões basins, where they comprise up to 20% of the stratigraphy. These basins contain extensive deposits of carbonate and evaporite, in addition to organic-rich shales and major hydrocarbon reservoirs. Here we show that large scale volatile generation followed sill emplacement in these lithologies. Thermal modeling demonstrates that contact metamorphism in the two basins could have generated 88,000 Gt CO_2_. In order to constrain the timing of gas generation, zircon from two sills has been dated by the U-Pb CA-ID-TIMS method, resulting in ^206^Pb/^238^U dates of 201.477 ± 0.062 Ma and 201.470 ± 0.089 Ma. Our findings demonstrate synchronicity between the intrusive phase and the end-Triassic mass extinction, and provide a quantified degassing scenario for one of the most dramatic time periods in the history of Earth.

## Introduction

The end-Triassic extinction (ETE) is one of the largest mass extinctions of the Phanerozoic. Both marine and terrestrial ecosystems were severely affected, and evidence from the fossil record indicates a total species loss of as much as 80%^1^. The ETE is associated with a 3–6‰ negative carbon isotope excursion (CIE) recorded in both marine and terrestrial records^2–6^, and by global warming of up to 3-4 °C (ref.^7^). Previous studies have temporally linked the ETE to the onset of the Central Atlantic Magmatic Province (CAMP^8^), leading to the hypothesis that CAMP volcanism triggered the end-Triassic crisis^4–6,8–11^. However, in all available stratigraphic sections, preserved lava flows post-date the ETE-marking CIE^4–6,12^, making this link questionable. Furthermore, carbon cycle models show that the magnitude of CO_2_ release from the CAMP lavas is not sufficient to explain the 3–6‰ negative CIE considering a mantle δ^13^C value of ~−5‰, and that a more ^12^C-enriched carbon source was potentially involved^1,13,14^.

A recent study^15^ used high precision U-Pb geochronology to demonstrate that the sub-volcanic phase of CAMP started 150 ± 38 ky before the oldest basalt flows, and showed that the emplacement of igneous intrusions in Brazil, Spain and Bolivia occurred synchronously with the ETE (201.564 ± 0.015 Ma^11^). This is significant as a large portion of the CAMP is preserved as sills^16^, which are particularly widespread in northern Brazil^17–22^. These sills are emplaced in the Amazonas and Solimões basins (and likely also in the Acre Basin^17^) and are estimated to ~5 × 10^5^ km^3^, which represents ca. 75% of the total volume of known CAMP sills^16^. The Amazonas and Solimões basins comprise a thick sedimentary sequence (up to 5 km thick^18^), including organic-rich shales, carbonates and evaporites^18–20^. Contact metamorphism and/or assimilation of host rock related to sill emplacement can lead to significant sediment-derived volatile generation if the host rocks are rich in organic matter and/or evaporites^23^. A causal link between the generation of isotopically light carbon from contact metamorphism and negative CIEs has been proposed for other global events such as the end-Guadalupian, end-Permian, end-Toarcian, and the Paleocene-Eocene^24–29^. The generated gases in the Brazilian basins could have included SO_2_, halogens, halocarbons and/or polycyclic aromatic hydrocarbons (PAHs), and it has been speculated that these gases were involved in triggering the ETE^26,30–35^. However, the distribution and ages of the sills in the Brazilian basins are poorly constrained, and the metamorphic effects on the sedimentary rocks have not been quantified.

Here we present data from seven deep (up to 3100 m) boreholes from the Amazonas and Solimões basins. We test the role of sill intrusions and contact metamorphism in the end-Triassic crisis by: 1) characterizing the extent of sill intrusions, their thicknesses and emplacement depths, 2) modelling the CO_2_ generation during contact metamorphism of the host sedimentary rocks, and 3) providing new constraints on the timing of sill emplacement by high-precision U-Pb zircon geochronology.

## The Solimões and Amazonas basins

**Figure 1:**
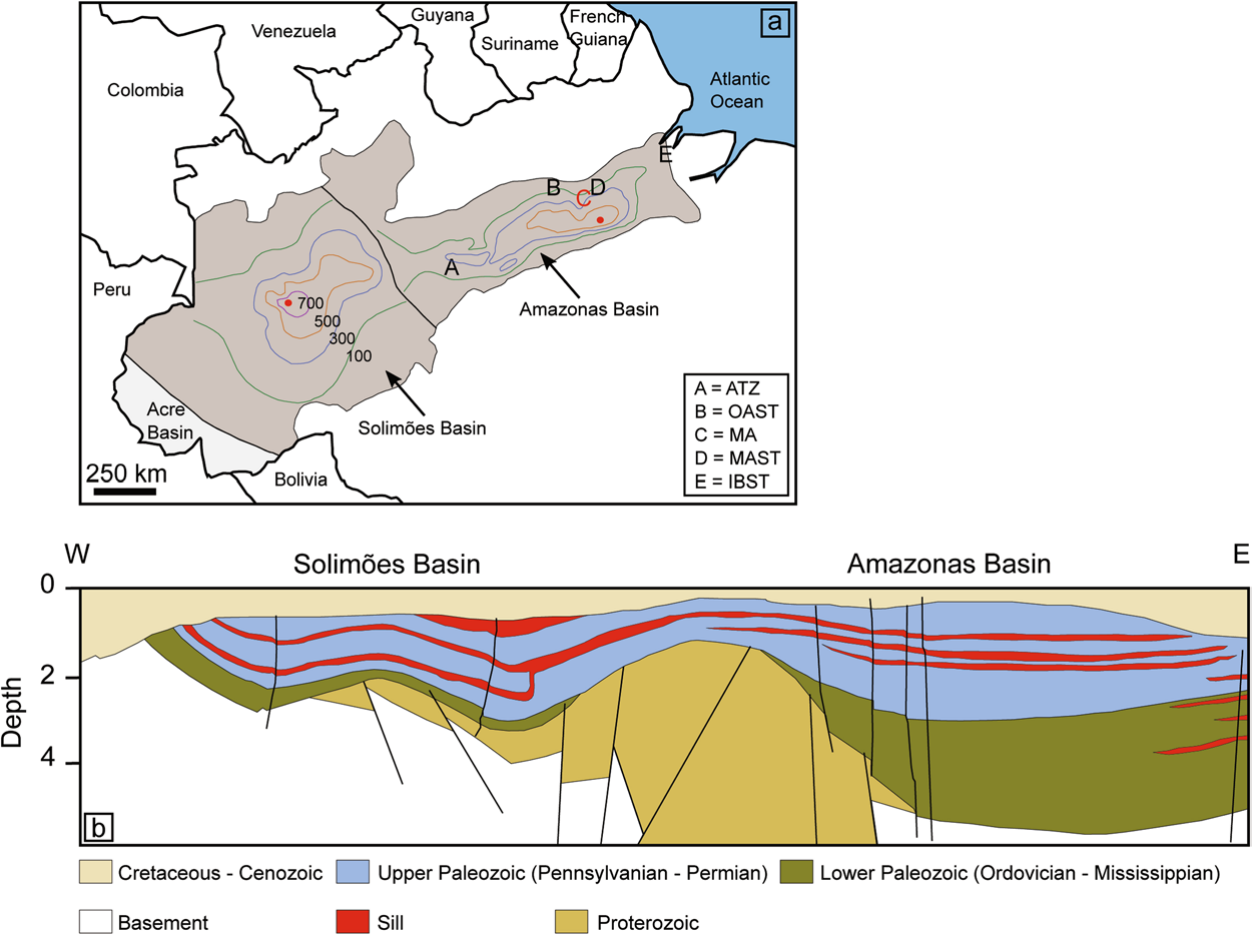
(**a**) Outlines of the Solimões and Amazonas sedimentary basins (after ref.^36^), located in northern Brazil. Colored contour lines represent the sill thickness distribution within the basins, and the red dots mark the maximum cumulative sill thickness^17,20^. The sill thickness peaks in the central parts of both basins, with 1038 m for the Solimões Basin and 915 m for the Amazonas Basin, and decreases toward the margins. Capital letters represent borehole locations. The sill from the Amazonas Basin dated in this study is from borehole MA (location C), which is marked in red. The map is manually redrawn based on Google Maps provided by Map data ©2017 Google. (**b**) Schematic cross-section of the Solimões and Amazonas basins (redrawn after ref.^69^) showing that sills are widespread in the Upper Paleozoic series. Note that sills within Lower Paleozoic sedimentary rocks are restricted to the eastern part of the Amazonas Basin.

The vast onshore Amazonas and Solimões sedimentary basins are located in northern Brazil (Fig. 1a), covering an area of more than 1 × 10^6^ km^2^ (ref.^20^). The sedimentary deposits are predominantly Paleozoic (Fig. 1b), and were intruded by sills during the emplacement of CAMP^19,36^. The Paleozoic series consist of a lower unit of Ordovician-Mississippian shales and sandstones, and an upper Pennsylvanian-Permian unit dominated by carbonate and evaporite deposits, the latter reaching up to 1600 m in thickness^18^. The lower unit includes deposits of black shale with high total organic carbon (TOC) concentrations (up to 8 wt.%^18,19,21^), while hydrocarbon reservoirs occur in both the upper and lower units^18,19^. Sills are widespread within the upper unit, extending continuously from the western margin of the Solimões Basin to the eastern margin of the Amazonas Basin (Fig. 1b). In the lower unit, sills are considered to be restricted to the eastern part of the Amazonas basin. The maximum cumulative sill thicknesses are present in the central parts of both basins, with 1038 m in the central Solimões Basin and 915 m in the central Amazonas Basin^20^, decreasing towards the margins (Fig. 1a).

## Results

### Log data and samples

**Figure 2:**
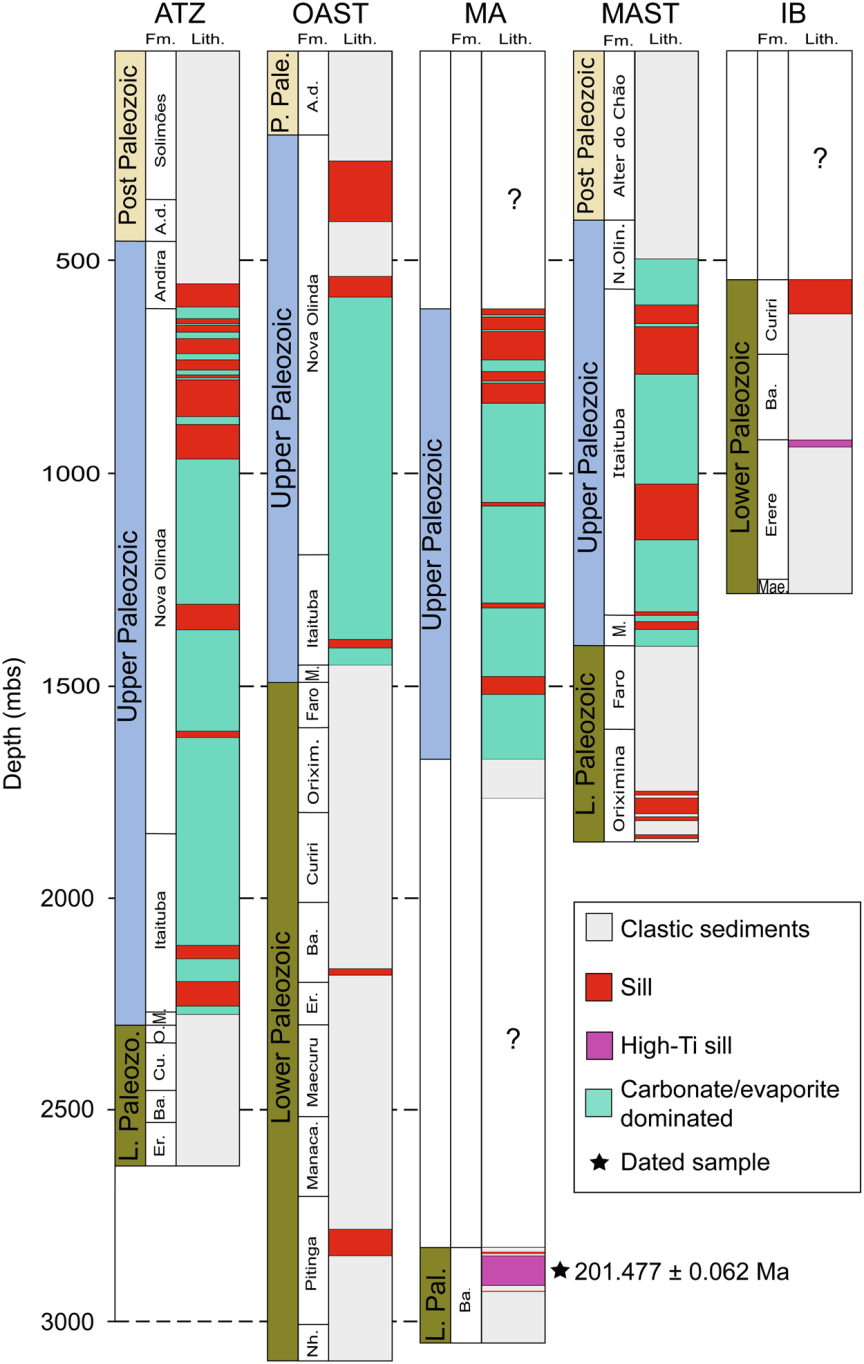
Representative schematic borehole logs (ordered from W to E) from the Amazonas Basin show that the majority of recovered sills have intruded the upper Paleozoic section, predominantly consisting of carbonates and evaporites. Available borehole locations are presented in Fig. 1a (note that the location for borehole IB is not known). Mbs. = meters below surface. Fm. = formation. Lith. = lithology. P. Pale. = Post Paleozoic. L. Pale./Paleozo. = Lower Paleozoic. A.d. = Alter do Chão. M. = Monte Alegre. Orixim./O. = Oriximiná. Ba. = Barreirinha. Er. = Ererê. Manaca. = Manacapuru. Nh. = Nhamundá. Cu. = Curiri. N. Olin. = Nova Olinda. Mae. = Maecuru.

A set of 32 dolerite samples and log data from seven boreholes were selected and provided by the National Petroleum Agency in Brazil and the HRT Brazilian Oil Company; one borehole from the Solimões Basin and the remaining six from the Amazonas Basin. Samples were available from the Solimões Basin borehole and all but one of the six Amazonas Basin boreholes. Log data were only available for five boreholes from the Amazonas Basin (Fig. 2). An overview of the studied boreholes is presented in Supplementary Table S1.

The seven boreholes comprise a total of 41 sills, and log-based thickness data are available for 39 sills (Supplementary Table S1). The cumulative sill thickness per borehole ranges from 91 to 482 m (mean of 308 m), representing up to 20% of the stratigraphy. The individual sill thicknesses vary significantly (3–143 m), however more than 70% of the sills are <60 m thick.

The majority of the sills (>70%) are located within the evaporite- and carbonate- dominated lithologies of the Nova Olinda, Itaituba and Monte Alegre formations of the Upper Paleozoic series (Fig. 2). Five sills are emplaced in black shale with potentially high TOC contents, including four sills (up to 70 m thick) in the Barreirinha formation (TOC up to 8 wt.%^18,19^) and one 62 m thick sill in the Pitinga formation (TOC up to 4 wt.%^19^). Three sills are present in the hydrocarbon-bearing^19^ Monte Alegre, Curiri and Ererê formations. The five remaining sills are emplaced in clastic sedimentary rocks of the Andirá and Oriximiná formations.

### U-Pb geochronology

Single zircon grains from two subsurface sill samples were analysed by the high-precision U-Pb CA-ID-TIMS technique (see Methods). Complete U-Pb data and date distribution plots are presented in Supplementary Table S2 and Figure S1, respectively. Weighted mean ^206^Pb/^238^U dates are calculated based on at least three youngest overlapping zircon analyses from each sample, with uncertainties reported at the 95% confidence level (2σ) and following the notation ±X/Y/Z Ma, where X is the internal (analytical) uncertainty in the absence of all external errors, Y incorporates the U-Pb tracer calibration error and Z includes the latter as well as the U decay constant errors^37^. When comparing U-Pb data produced using the same isotopic tracer, (refs^11,15^ and this study), only X needs to be considered.

Five zircon grains analysed from sample 1-MA-1-PA-2883.18 (Amazonas Basin) yielded a cluster of statistically coherent analyses with a weighted mean ^206^Pb/^238^U age of 201.477 ± 0.062/0.11/0.24 Ma and a mean square of weighted deviates (MSWD) of 1.7. In contrast, nine zircon grains analysed from sample Amostra 8 (Solimões Basin) produced a wide range of ^206^Pb/^238^U dates from 2803.7 ± 4.4 Ma (z3) to 201.42 ± 0.19 Ma (z5), reflecting an abundance of xenocrystic zircons in this sill. The three youngest analyses, however, comprise a tight cluster with a weighted mean ^206^Pb/^238^U date of 201.470 ± 0.089/0.13/0.25 Ma (MSWD = 0.16).

### Thermal modelling of gas generation around sills

Input data (Supplementary Tables S3–S5) for the numerical thermal sill model are based on boreholes ATZ, OAST and MAST (Fig. 2) from the Amazonas basin (Fig. 1a; location A, B and D respectively). These boreholes were selected as they comprise the most complete stratigraphy among the five boreholes with available log information, minimizing the uncertainties in the interpretation of the depositional history of the basin. Furthermore, in the Amazonas Basin sills are present in both the upper- and lower Paleozoic sedimentary series (Fig. 1b), thus combined these three boreholes give the full extent of sill emplacement. Present day TOC and vitrinite reflectance values are included when available (Supplementary Table S6).

The relative timing of emplacement among the individual sills is poorly constrained, however U-Pb geochronology demonstrates that sill emplacement was not simultaneous (see discussion). If the sills are closely spaced, as observed for borehole ATZ and MAST, selecting simultaneous sill emplacement in the model would result in a higher maximum temperature within the aureoles^38^, which could lead to overestimation of the CO_2_ production (Supplementary Figure S2). The timescale of sill cooling is generally in the order of 10 s to 1000 s of years^39^, depending on the sill thickness. To produce a more conservative estimation, an emplacement scenario with individual sill emplacement every 10,000 years was used in the model. We stress that this modeled timing of sill emplacement is not constrained by our U-Pb geochronology, but is chosen to prevent overestimation of the thermal effects from closely spaced sills.

CO_2_ generation from pure carbonate generally occurs at temperatures above 950 °C, thus impure carbonates (i.e. marls) are required to generate substantial CO_2_ volumes during contact metamorphism^28^. In the model, the mineral composition of the carbonate can be chosen as 1) marl, 2) dolomite or 3) dolomite/evaporite mix. Geochemical and petrological analyses of carbonates from the Amazonas Basin demonstrate that they have varying compositions of SiO_2_ (up to 9 wt.%) and modal proportions of quartz up to 24% (Egberto Pereira, pers. comm.); i.e. they are not pure carbonates. The carbonate-dominated deposits were selected as marl in the model, however note that the carbonates are generally interlayered with clastic sedimentary rocks and evaporites.

### Borehole ATZ

Borehole ATZ comprises 2618 m of sedimentary rocks and 12 individual sills (Fig. 2). The sills are restricted to the upper Paleozoic section (Nova Olinda and Itaituba formations), dominated by carbonates and evaporites (Supplementary Table S1). As heating of pure evaporite would not produce CO_2_, the presence of evaporite has been accounted for by separating the five thickest halite layers (up to ~80 m) from the Itaituba and Nova Olinda sediments where the carbonates are present (Supplementary Table S3).

**Figure 3:**
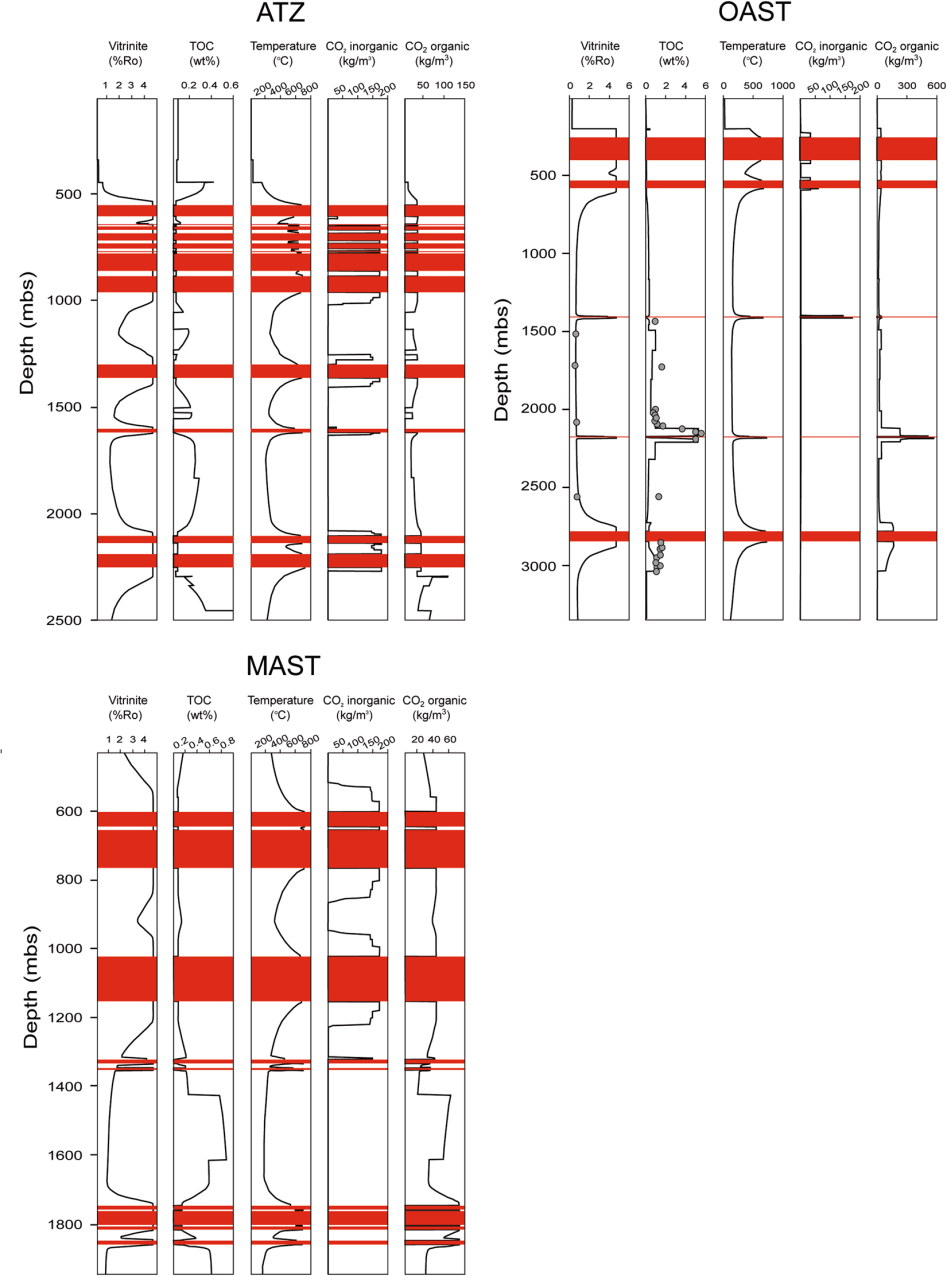
Modeled thermal effects of sills emplaced in Amazonas Basin sedimentary rocks at 1100 °C in intervals of 10,000 years, based on data from borehole ATZ, OAST and MAST. The temperature and vintrinite reflectance values (%Ro) increase, whereas the total inorganic carbon (TOC) content decreases toward each sill. Present day TOC and %Ro values (grey dots) generally follow the modeled values. Organic CO_2_ is generated in all aureoles, whereas generation of inorganic CO_2_ is restricted to aureoles within carbonate-bearing lithologies (upper Paleozoic series). For borehole ATZ, all sills are emplaced in the upper Paleozoic section, whereas for boreholes OAST and MAST, sills are emplaced in both upper and lower Paleozoic sedimentary rocks.

The modeled metamorphic effects of each individual sill in borehole ATZ are shown in Fig. 3. The temperature rises from background values and reaches a maximum at the innermost aureole around each sill. The TOC content decreases towards each sill, and correspondingly, the vitrinite reflectance values (%Ro) increase. Both organic and inorganic CO_2_ is generated in aureoles around all of the 12 sills (Fig. 3).

**Figure 4:**
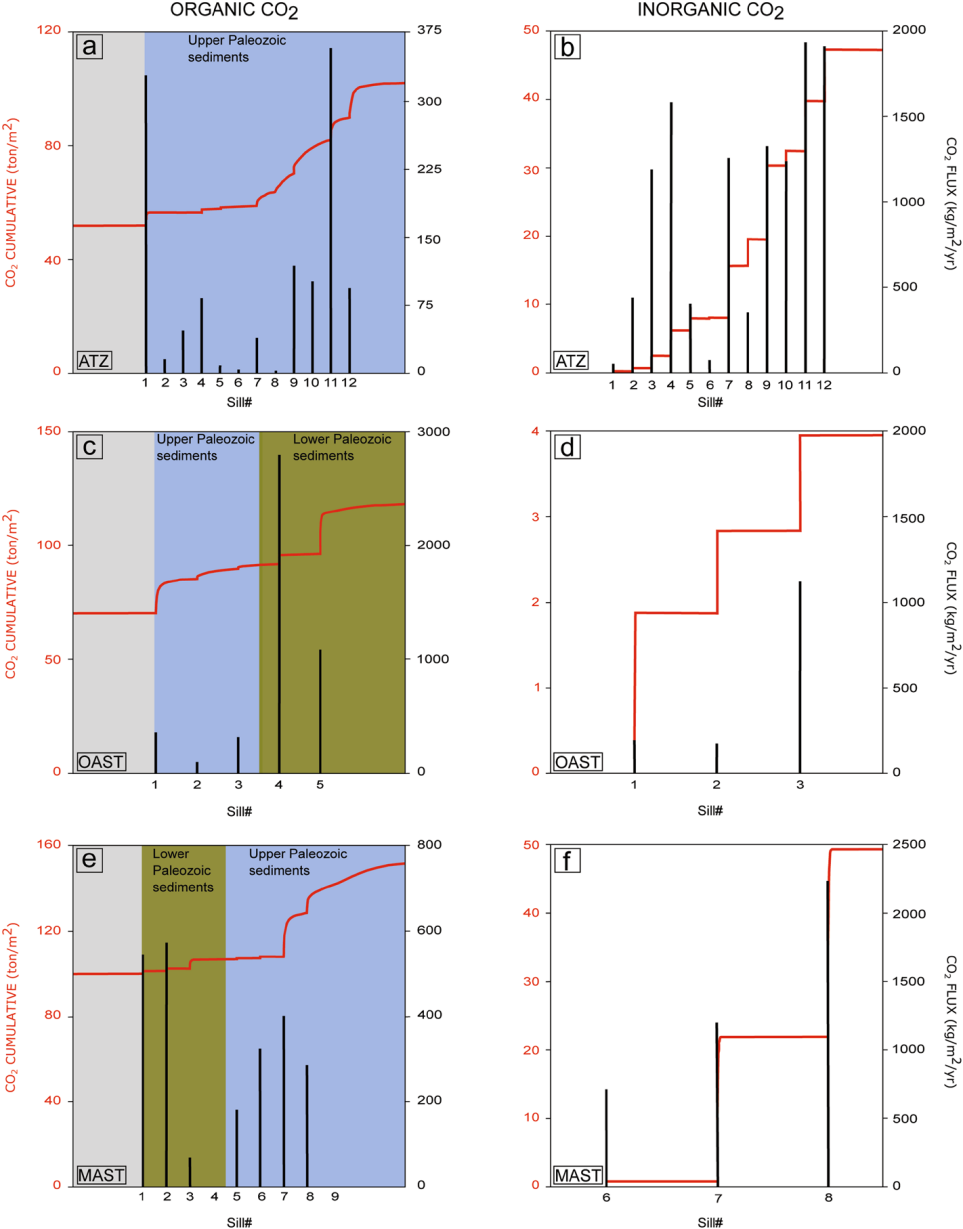
Modeled cumulative CO_2_ production (red lines) and peak fluxes (black lines) per sill based on data from borehole ATZ (Fig. a; organic, Fig. b; inorganic), OAST (Fig. c; organic, Fig. d; inorganic) and MAST (Fig. e; organic, Fig. f; inorganic). (**a**) The organic CO_2_ production increases from the background value (52 ton/m^2^) after the first sill emplacement, and peaks at 102 ton/m^2^ after the last. The total organic CO_2_ production due to sill heating is 50 ton/m^2^. Peak organic CO_2_ fluxes vary from 2 to 363 kg/m^2^/yr. (**b**) The total inorganic CO_2_ production is 47 ton/m^2^. Peak inorganic CO_2_ fluxes vary from 52 to 1936 kg/m^2^/yr. (**c**) The organic CO_2_ production increases from the background value (70 ton/m^2^) after the first sill emplacement, and peaks at 118 ton/m^2^ after the last. The total organic CO_2_ production due to sill heating is 48 ton/m^2^. For the three sills emplaced in the upper Paleozoic series, the organic CO_2_ production is 22 ton/m^2^, whereas for the two sills emplaced in lower Paleozoic sedimentary rocks, the organic CO_2_ production is 27 ton/m^2^. Peak organic CO_2_ fluxes are significantly lower for sills emplaced in upper Paleozoic sedimentary rocks (95 to 356 kg/m^2^/yr) compared to sills emplaced in the lower Paleozoic ones (up to 2794 kg/m^2^/yr). (**d**) The total inorganic CO_2_ production is 4 ton/m^2^. There is no inorganic CO_2_ production for the two sills emplaced in lower Paleozoic sedimentary rocks. Peak inorganic CO_2_ fluxes vary from 190 to 1122 kg/m^2^/yr. (**e**) The organic CO_2_ production increases from the background value (100 ton/m^2^) after the first sill emplacement, and peaks at 154 ton/m^2^ after the last. The total organic CO_2_ production due to sill heating is 54 ton/m^2^. The organic CO_2_ production is 47 and 7 ton/m^2^ from contact metamorphism of upper- and lower Paleozoic sections, respectively. Peak organic CO_2_ fluxes vary from 64 to 573 kg/m^2^/yr. Note that there are no peaks for sill #4 and #9. Sills that are closely spaced could affect the same sedimentary rocks. As sill #3 and #8 are thicker and were emplaced prior to the respective sills #4 and #9, the CO_2_ was already generated when sill #4 and #9 were emplaced. (**f**) The total inorganic CO_2_ production is 49 ton/m^2^. There is no inorganic CO_2_ production for the four sills emplaced in lower Paleozoic sedimentary rocks. For the sills emplaced in the in upper Paleozoic series, the peak inorganic CO_2_ fluxes vary from 711 to 2235 kg/m^2^/yr. Note that there are no peaks for sill #5 and #9. Sill #5 is close to the lower Paleozoic section, and affects more or less only clastic sedimentary rocks (no marl), whereas sill #8, which is thicker and were emplaced prior to sill #9, already affected the sedimentary rocks surrounding sill #9.

The cumulative CO_2_ production for borehole ATZ is 97 ton/m^2^, including 50 ton/m^2^ organic CO_2_ (Fig. 4a) and 47 ton/m^2^ limestone-derived inorganic CO_2_ (Fig. 4b). The organic CO_2_ production is calculated by subtracting the CO_2_ value from background maturation (52 ton/m^2^) from the peak CO_2_ value after sill emplacement (102 ton/m^2^). See Supplementary Table S7 for full overview of peak organic and inorganic CO_2_ fluxes (kg/m^2^ per year) per sill emplacement.

### Borehole OAST

Borehole OAST comprises 3092 m of sedimentary rocks and 5 individual sills emplaced within both upper and lower Paleozoic series (Fig. 2). The sills in the lower Paleozoic section are emplaced in the Barreirinha and Pitinga formations, with initial TOC contents of 8 and 2.3 wt.% respectively (Supplementary Table S4). The modeled temperature, TOC and vitrinite reflectance values follow the same trend as for borehole ATZ (Fig. 3). For borehole OAST, present day TOC and vitrinite reflectance values are included in the model, and there is generally a good fit with the modeled values. Present day TOC values for the Itaituba (1430 m), Oriximina (1722 m) and Manacapuru formations (2559 m) are higher than the modeled values. As there is only a single measurement per formation, these values might not be representative for the entire respective sedimentary formations. Present day TOC values are also significantly higher than the modeled values within Pitinga formation sedimentary rocks, immediately below the stratigraphically lowest sill (2852–2930 m depth; TOC: 1.43–1.61 wt.%). Such high TOC values within the contact aureole zone (%Ro >1; ref.^29^) seem unrealistic, thus we attribute the elevated values to poor depth control of the measured cuttings.

Organic CO_2_ is generated in aureoles around all sills, whereas inorganic CO_2_ is restricted to aureoles within carbonate-bearing lithologies in the upper Paleozoic section (Fig. 3). The cumulative CO_2_ production modeled for borehole OAST is 52 ton/m^2^, including 48 ton/m^2^ organic CO_2_ (Fig. 4c) and 4 ton/m^2^ limestone-derived inorganic CO_2_ (Fig. 4d).

### Borehole MAST

Borehole MAST comprises 1863 m of sedimentary rocks and 9 individual sills. 5 sills are emplaced within the upper Paleozoic section (Itaituba and Monte Alegre formations; dominated by carbonate, evaporite is more or less absent – Supplementary Table S1), and 4 sills are emplaced in clastic sedimentary rocks of the lower Paleozoic section (Oriximiná Formation) (Fig. 2).

The modeled temperature, TOC and vitrinite reflectance values follow the same trend as for borehole ATZ and OAST. Organic CO_2_ is generated in aureoles around all sills, whereas inorganic CO_2_ is restricted to aureoles within the carbonate-bearing upper Paleozoic section (Fig. 3). The cumulative CO_2_ production for borehole MAST is 103 ton/m^2^, including 54 ton/m^2^ organic CO_2_ (Fig. 4e) and 49 ton/m^2^ limestone-derived inorganic CO_2_ (Fig. 4f). Note that he bulk of the organic CO_2_ (47 ton/m^2^; ~90%) is generated in aureoles within the upper Paleozoic section.

## Discussion

Basin cross-sections show that CAMP sills in Brazil are widespread in the upper Paleozoic section, extending continuously from the western margin of the Solimões Basin to the eastern margin of the Amazonas Basin (Fig. 1b). In the Solimões Basin, sills are only present in the upper Paleozoic series, however in the Amazonas Basin sills are also present in lower Paleozoic sedimentary rocks in the eastern part of the basin (Fig. 1b). The sill thickness distribution (Fig. 1a) does not provide information about the emplacement depths of the sills, however the Amazonas Basin boreholes (Fig. 2) demonstrate that the majority (>70%) of recovered sills intruded the upper Paleozoic section (Supplementary Table S1). Hence, the contour lines for the Amazonas Basin in Fig. 1a predominantly represent sills emplaced in the upper Paleozoic section. The observations that 1) sills are distributed throughout a large area of both basins (Fig. 1a) and 2) the majority of sills recovered by the boreholes are located within upper Paleozoic sedimentary rocks (Fig. 2), suggest that contact metamorphism of upper Paleozoic sedimentary rocks was not a localized process but rather occurred on a large scale.

Boreholes ATZ and MAST comprise multiple sills in the upper Paleozoic section, and the total sill thickness for these boreholes (482 and 367 m respectively; Supplementary Table S1) could be considered average values as the maximum cumulative sill thickness recorded is 1038 m^20^. The amount of inorganic CO_2_ generated from decarbonation reactions depends on the amount of impure carbonate present. Borehole ATZ recovered ~1200 m of carbonate- and evaporite-dominated deposits with a high evaporite to carbonate ratio, whereas borehole MAST recovered considerably less of these deposits (~700 m), but including only 3 m of evaporite (Supplementary Table S1). Carbonate- and evaporite-dominated lithologies in the Solimões and Amazonas basins reach up to 1300 and 1600 m (ref.^18^) respectively, however the ratio of carbonate to evaporite within these deposits is poorly constrained. The magnitude and ratio of organic vs. inorganic CO_2_ modeled for upper Paleozoic sedimentary rocks from boreholes ATZ (50 and 47 ton/m^2^ organic and inorganic CO_2_, respectively) and MAST (47 and 49 ton/m^2^ organic and inorganic CO_2_, respectively) are more or less identical, indicating that these results could reflect an average value. The upper Paleozoic sections affected by sill intrusions in borehole ATZ and MAST are therefore considered to be representative for the Amazonas and Solimões basins. Paleozoic sedimentary rocks are present within an area of 500,000 km^2^ in the Amazonas Basin^22^ and 400,000 km^2^ in the Solimões Basin^18^. The modeled CO_2_ value of ~97 ton/m^2^ generated from contact metamorphism of upper Paleozoic sedimentary rocks in boreholes ATZ and MAST is therefore extrapolated to a total area of 900,000 km^2^. This yields a production of 88,000 Gt CO_2_, including 44,000 Gt organic and 44,000 Gt inorganic CO_2_. The CO_2_ produced by CAMP sills intruding the Acre basin is not taken into account here, first because of the lack of available boreholes to base the model on and second because it is not entirely clear if those sills are CAMP-related or not.

The lower Paleozoic unit in the Solimões and Amazonas basins includes black shale with high TOC contents (up to 8 wt.%^18,19,21^). Borehole OAST comprises two sills that are emplaced in TOC-rich shales within the Pitinga (2.3 wt.% TOC) and Barreirinha (8 wt.% TOC) formations (Fig. 2; Supplementary Table S4). The thermal modeling estimates that ~27 ton CO_2_/m^2^ can be derived from contact metamorphism of these sedimentary rocks alone (Fig. 4c), which represents more than 50% of the total organic CO_2_ production (48 ton/m^2^) modeled for borehole OAST. While the sill found in the Barreirinha formation in this borehole is only 3 m thick (Supplementary Table S7), the presence of multiple, and much thicker sills (up to 70 m) within this formation is found by several other boreholes (e.g. borehole MA; Fig. 2). The generation of organic CO_2_ from contact metamorphism of Barreinrinha shales is therefore likely to be significantly higher than estimated by the thermal sill model for borehole OAST. Lower Paleozoic organic-rich black shales (Jandiatuba formation; TOC up to 8 wt.%^18,21^) were also thermally affected by sills in the western part of Solimões Basin, where the deepest sills are close to the shales. Sill emplacement has been linked to the majority of petroleum reservoirs in the Solimões Basin^18,20,21,40^. As sill emplacement in lower Paleozoic sedimentary rocks was only localized and not widespread (Fig. 1b), the CO_2_ generated from contact metamorphism of these rocks has not been added to the basin scale estimate. However, we stress that lower Paleozoic organic-rich deposits were heated by sills in both basins, and that this also contributed to the total volatile load.

It is important to note that the thermal model does not account for interaction between sills and pre-existing accumulations of hydrocarbons, which could lead to enhanced gas production^41^. In the Amazonas Basin, hydrocarbon generation was initiated in the Carboniferous and was completed in the early Triassic^19^. This means that the Amazonas Basin was petroleum-bearing prior to sill emplacement in the late-Triassic, and previous studies have shown that the sill emplacement led to a peak in gas generation from reservoir heating^18–20^. Secondary hydrocarbon cracking due to sill heating has also been proposed for the Solimões Basin^20,42^.

Contact metamorphism of evaporites can lead to the generation of gases such as SO_2_ and halocarbons (e.g. CH_3_Cl)^26,28,38^. Additionally, volatiles from evaporites can be incorporated into the magma by assimilation, increasing the concentration of dissolved volatiles^43–45^. The upper Paleozoic sedimentary units in the Solimões and Amazonas basins comprise thick evaporite deposits; the boreholes studied here have recovered up to ~500 m of anhydrite and halite (Supplementary Table S1). The thermal sill model is a general purpose model which only considers decarbonation reactions^46^, thus potential evaporite-derived volatile generation is not quantified here. However, considering that sills are widespread in evaporites, it is likely that sill-evaporite interaction contributed to the total volatile load generated in the Solimões and Amazonas basins.

Gases generated by thermogenic processes are likely to be partly released to the atmosphere through explosion pipes or fractures, and partly trapped in the host sedimentary rocks. Rapid gas generation leads to overpressure buildup, which results in hydrofracturing and/or pipe formation, especially in shales, limestones and evaporites^47^. Hydrothermal vents and breccia pipes are well documented in other sedimentary basins intruded by sills, such as the Karoo Basin in South Africa^25^, the Tunguska Basin in Siberia^26,48^ and the Vøring and Møre Basins in the Northeast Atlantic^24^. Evidence for explosion pipes in the Amazonas and Solimões basins have not been documented, probably due to the dense vegetation and sedimentary cover.

Carbon cycle perturbations in the latest Triassic and earliest Jurassic are characterized by three negative carbon isotope excursions (CIEs), separated by positive δ^13^C trends^6,49–51^. Note that these three CIEs have traditionally been termed “precursor”, “initial” and “main”, however we will follow the new nomenclature recently proposed by ref.^51^. The first disruption of the carbon cycle is marked by the 2–3‰^49,50^ negative Marshi CIE, which predates the 3–6‰^2–6^ negative Spelae CIE by 100–200 ky^4^. The 2–3‰^3^ negative Tilmanni CIE represents the third excursion and is characterized by a prolonged period (120 ky; ref.^52^) of decreasing δ^13^C before stabilization of the carbon cycle takes place in the earliest Jurassic^4^.

**Figure 5:**
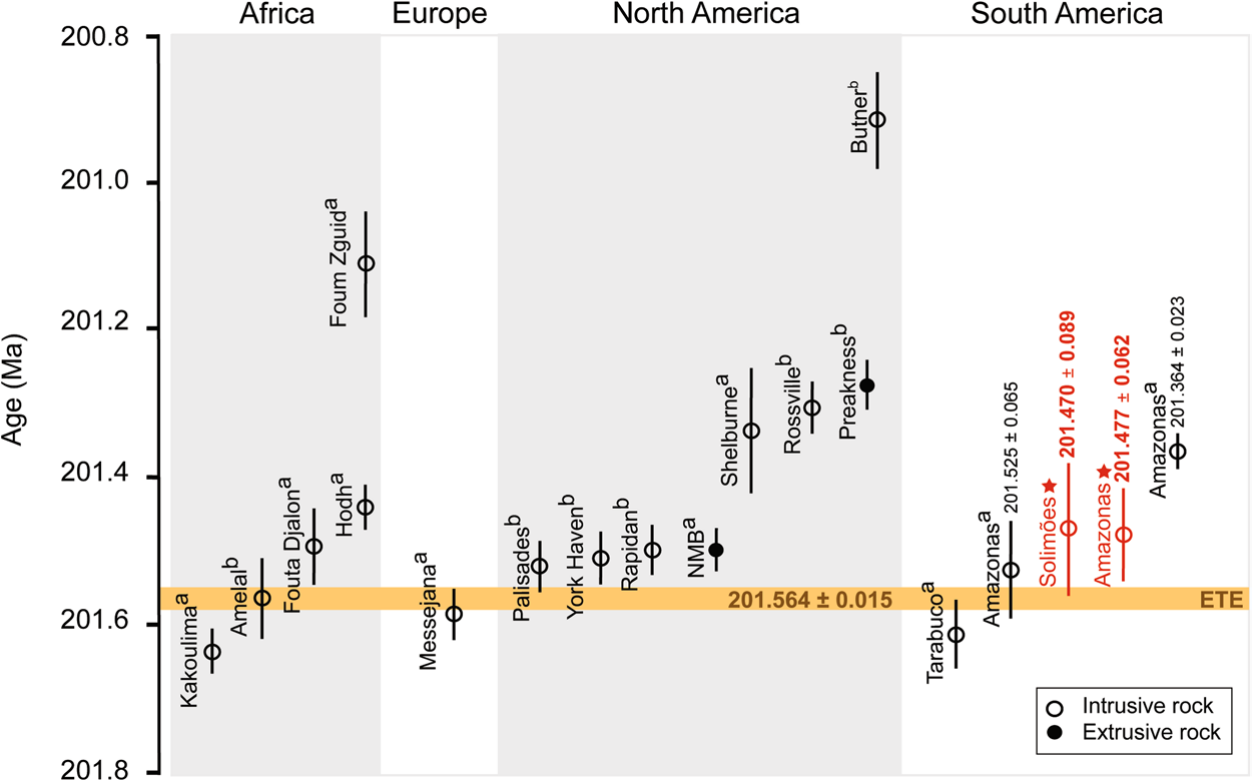
Compilation of available high precision U-Pb ages of Central Atlantic Magmatic Province (CAMP) intrusive (open circle) and extrusive (filled circle) rocks from North America, South America, Europe and Africa. The orange horizontal line represents a calculated U-Pb age of the end-Triassic extinction (ETE)^11^. The oldest age for CAMP magmatism is 201.635 ± 0.029 Ma, dated from the Kakoulima intrusion in Guinea^15^, attesting for the occurrence of magmatism prior to the ETE. Two sills from the Solimões and Amazonas basins overlap with the age of the ETE. Sills dated in this study are marked in red and by a star symbol. The sill from the Solimões Basin has an U-Pb age of 201.470 ± 0.089 Ma whereas the Amazonas sill is dated to 201.477 ± 0.062 Ma. The remaining U-Pb ages are from: (**a**) ref.^15^ and (**b**) ref.^11^. Note that the age for the North Mountain Basalt (abbreviated as NMB) is an average age of ages from refs^9,11,15^.

The ETE is generally considered to coincide with the (“initial”) Spelae CIE^4–6,10^. However, stratigraphic records show that major biotic changes, including the extinction of plants and bivalves, correlate with the earlier (“precursor”) Marshi CIE^31,49,51^. The oldest known CAMP basalts in North America and Morocco occur marginally after the Spelae CIE in stratigraphic sections^4–6,12^, which means that (at least the preserved) extrusive component of CAMP post-dates the ETE. On the other hand, high precision U-Pb geochronology suggests that intrusive CAMP magmatism, represented by the Kakoulima intrusion in Guinea, started 150 ± 38 ky before the oldest basalt eruptions^15^ (Fig. 5). Based on an astronomical tuned section in the Newark Basin (USA), anchored by high precision U-Pb dating of CAMP basalts, the age of the ETE has been estimated to 201.564 ± 0.015 Ma (ref.^11^). Two of the dated sills from the Solimões and Amazonas basins have U-Pb ages of 201.525 ± 0.065 Ma (ref.^15^) and 201.470 ± 0.089 Ma (this study) and overlap therefore with the ETE, whereas the high-Ti sill from the Amazonas Basin with a U-Pb age of 201.477 ± 0.062 (this study) slightly post-dates the ETE (Fig. 5). A recent study^51^ proposed a new correlation for the T-J boundary sections, suggesting that the negative CIE marking the ETE underlying the oldest basalts in North America and Morocco is in fact the Marshi CIE and not the Spelae CIE as previously assumed. This indicates that the intrusives overlapping with the ETE (Fig. 5), including the two oldest Brazilian sills, could correlate with the Marshi CIE, and that the youngest sill from the Amazonas Basin (201.364 ± 0.023 Ma; ref.^15^) occur synchronously with the Spelae CIE.

Carbon cycle models^1,13,14^ have focused on the 3–6‰ negative Spelae CIE, which they link to input of ^13^C-depleted carbon, and agree that the magnitude of CO_2_ release from the CAMP lavas is not sufficient to explain this CIE considering a mantle δ^13^C value of −5‰. These models suggest either (1) a combined release of ~30,000 Gt volcanic CO_2_ from CAMP (δ^13^C = −5‰) and ~18,000 Gt CO_2_ derived from methane hydrates (δ^13^C = −60‰)^1^, (2) release of ~78,000 Gt volcanic CO_2_ from CAMP with an isotopic composition of −20‰^13^, or (3) that highly depleted carbon (δ^13^C = −70‰) was involved^14^, i.e. these models suggest either dissociation of methane hydrates (or other methane pools) or that the mantle had an anomalously low δ^13^C signature. To date, there is no evidence that confirms either of these hypotheses. Here we show that 88,000 Gt CO_2_ could have been generated during contact metamorphism in the Solimões and Amazonas basins, with a more or less equal contribution of organic and inorganic CO_2_. δ^13^C values of organic matter and carbonates in Amazonas Basin range from −27 to −31‰ and ~0 to 4‰, respectively (Egberto Pereira, pers. comm.). A simple mass balance calculation assuming 44,000 organic CO_2_ with a δ^13^C value of −29‰ and 44,000 inorganic CO_2_ with a δ^13^C value of 2‰, yields a δ^13^C value of −13.5‰ for the 88,000 Gt CO_2_. Assuming 100% degassing, a mass balance calculation with end-Triassic boundary conditions^1,10^ suggests a negative CIE of ~−3‰, which is of a similar magnitude as the Marshi CIE (−2–3‰^49,50^). Estimating the amount of thermogenic gases that are released vs. trapped in sedimentary basins is challenging, however 100% degassing is regarded as unrealistic. Contact metamorphism of lower Paleozoic sedimentary rocks (with TOC up to 8 wt.%^18,19,21^) has not been taken into account, thus the contribution of organic carbon is probably significantly underestimated. The estimated δ^13^C drop of ~−3‰ should therefore be considered with caution.

The youngest sill from the Amazonas Basin (201.364 ± 0.023 Ma; ref.^15^) is characterized by high titanium content (following a threshold of TiO_2_ >2 wt.% for high-Ti CAMP rocks^8^), a geochemical feature shown by a small percentage of CAMP rocks, both in Brazil and across the CAMP province^53–57^. This high-Ti sill crops out in the northeastern part of the Amazonas Basin^15^. Three of the sills from this study are defined as high-Ti, including the sill that slightly post-dates the ETE (201.477 ± 0.062 Ma; Fig. 5), and are recovered by boreholes IBST, MA and IB. As shown in Fig. 2, the high-Ti sills from boreholes IB and MA are emplaced in the lower Paleozoic unit. While a borehole log for IBST is not available, sills are known to intrude the lower Paleozoic section in the eastern part of the Amazonas Basin where this borehole is located (Fig. 1a; location E). Although outcrops and geochemical data from CAMP rocks in Brazil are limited, it appears that the occurrence of the high-Ti magma type in Brazil is restricted to the eastern part of the Amazonas Basin, within the lower Paleozoic series including black shales with high TOC concentrations. The emplacement of high-Ti sills in the Amazonas Basin could therefore have released later pulses of predominantly organic-derived CO_2_.

In addition to CO_2_ degassing, significant release of evaporite-derived S- and Cl-bearing compounds could have occurred from the Solimões and Amazonas basins. This could either be derived from direct release from contact metamorphism and/or by assimilation of the evaporites, which would increase the volatile concentrations of the CAMP magmas in these basins. It has been suggested that the release of such gases could explain the severe terrestrial stress associated with the ETE through acid rain and atmospheric pollution^26,30–35^. We suggest a pulsed sill emplacement scenario in which thermogenic gases generated by the earliest sills in Brazil contributed to triggering the ETE and the coinciding Marshi CIE. The Spelae CIE could reflect a later pulse of sill emplacement (high-Ti sills within lower Paleozoic sedimentary rocks) and release of thermogenic organic carbon. Future carbon cycle models of the end-Triassic interval should aim to include the Marshi CIE, while the environmental effects of a combined release of inorganic and organic carbon needs to be tested.

## Methods

### U-Pb geochronology

Drill cores and cuttings from six intrusive sill samples from the Amazonas and Solimões basins were processed by standard crushing, as well as magnetic and density separation techniques; only two yielded zircon grains for dating. Zircon analysis by the U-Pb CA-ID-TIMS technique followed the detailed analytical procedures described in ref.^58^. Pre-treatment of zircon by chemical abrasion^59^ designed to mitigate the effects of radiation-induced Pb loss, involved thermal annealing at 900 °C for 60 hours and leaching in concentrated HF at 210 °C for 12 hours. The EARTHTIME ET535 mixed ^205^Pb-^233^U-^235^U tracer^60,61^ was used in the analyses and isotopic measurements were made on either a VG Sector 54 or an Isotopx X62 multi-collector, solid-source, mass spectrometer equipped with Daly photomultiplier ion-counting systems at the Massachusetts Institute of Technology. Reduction of mass spectrometric data, as well as calculation of dates and uncertainties used the Tripoli and U-Pb_Redux software and associated algorithms^62,63^.

### Thermal sill model

Quantification of aureole processes was performed using a one-dimensional (1D) numerical model, Silli 1.0, which can be applied to study the thermal effects of sill intrusions in sedimentary basins globally^46^ The model uses heat conduction to transport heat away from the cooling sills. It also accounts for the sequential deposition of sedimentary layers through time, erosion, latent heat effects and gas generation by decarbonation reactions and hydrocarbon maturation. Although the effects of fluids flow may be significant, such as the perturbation of the thermal structure by heat advection, overpressure generation, vent formation and transport of hydrocarbons, these effects are largely limited to sill edges where fluid focusing occurs^47,64–66^. Therefore, the thermal structure in most regions around a sill can be reasonably approximated by thermal conduction.

Based on the depositional age of the basin lithologies, each sedimentary layer is deposited sequentially (including eroded layers), and the sedimentation rate is determined by layer thicknesses and top ages. The temperature within the sedimentary column is computed by applying fixed temperatures at the top and bottom of each layer, which are calculated from the estimated paleo-geotherm and energy diffusion equations^46^. The model assumes instant sill emplacement at a specified time.

Organic matter present in sedimentary rocks is thermally degraded at relatively high temperatures, thereby reducing TOC content. Vitrinite reflectance, a widely used indicator of thermal maturity, is calculated by the Easy%Ro method^67^. Organic CO_2_ generated from breakdown of organic matter is calculated from the difference in TOC content before and after sill emplacement. The amount of inorganic carbon released during decarbonation reactions is based on interpolation on the pressure-temperature phase diagrams generated by Perple_X using pre-defined rock geochemistry for marls^68^.

### Data Availability Statement

All datasets generated and/or analyzed during this study are included in this published article.

## Electronic supplementary material

Supplementary Information
