# Supplementary material for: Large-scale sill emplacement in Brazil as a trigger for the end-Triassic crisis
Source: Sci Rep. 2018 Jan 9;8:141. doi: 10.1038/s41598-017-18629-8 (PMC5760721; doi:10.1038/s41598-017-18629-8)
Supplement: Supplementary file 1 — Supplementary Information [file 41598_2017_18629_MOESM1_ESM.pdf]

Supplementary Information

Large-scale sill emplacement in Brazil as a trigger for the end-Triassic crisis

Thea H. Heimdal[1*], Henrik. H. Svensen[1], Jahandar Ramezani[2], Karthik Iyer[3,4], Egberto Pereira[5], René Rodrigues[5], Morgan T. Jones[1], Sara Callegaro[1]

[1]Centre for Earth Evolution and Dynamics (CEED), University of Oslo, PO Box 1047, Blindern, NO-0316 Oslo, Norway

[2]Department of Earth, Atmospheric and Planetary Sciences, Massachusetts Institute of Technology, Cambridge, MA 02139, USA

[3]GeoModelling Solutions GmbH, Zurich, Switzerland

[4]GEOMAR, Helmholtz Institute for Ocean Research, Kiel

[5]Department of Stratigraphy and Paleontology, Rio de Janeiro State University, Rio de Janeiro, Brazil

*Corresponding author: Thea Hatlen Heimdal. Address: Centre for Earth Evolution and Dynamics (CEED), University of Oslo, PO Box 1047, Blindern, NO-0316 Oslo, Norway. Tel: +47 41548103. E-mail address: t.h.heimdal@geo.uio.no.

Supplementary Figure S1

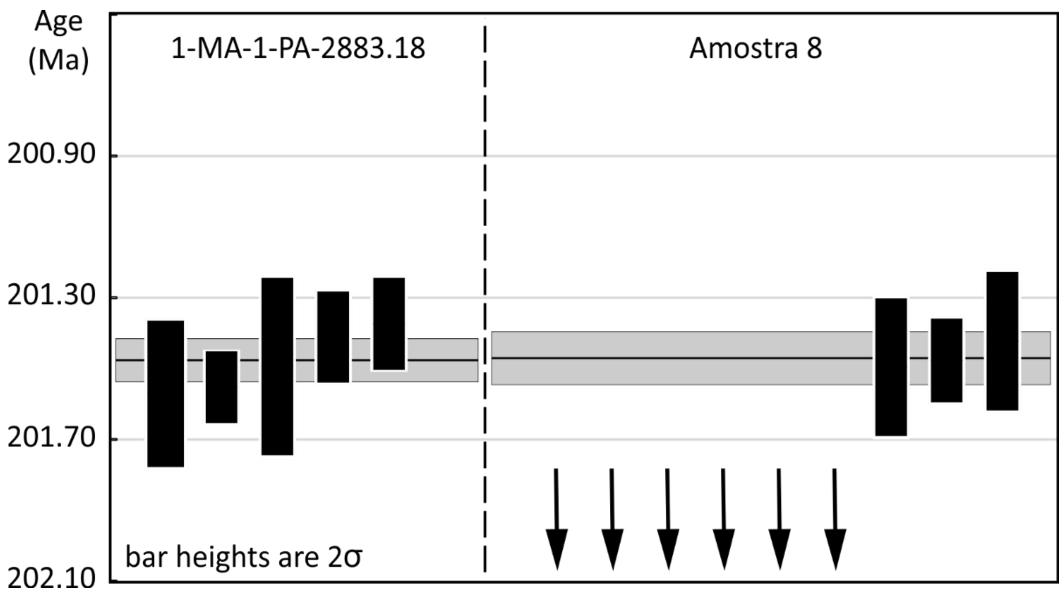

Date distribution plot of analysed zircons from the subsurface sills in the Solimões and Amazonas basins of Northern Brazil. Bar heights are proportional to 2σ analytical uncertainty of individual zircon analyses. Horizontal line and shaded envelope represent the calculated weighted mean $^{206}Pb/^{238}U$ dates and their 2σ internal error (X). These are 201.477 ± 0.062 Ma for sample 1-MA-1-PA-2883.18 (Amazonas Basin) and 201.470 ± 0.089 Ma for sample Amostra 8 (Solimões Basin). Arrows signify older xenocystic analyses that plot outside the diagram. See Table S2 for complete U-Pb data.

Supplementary Figure S2

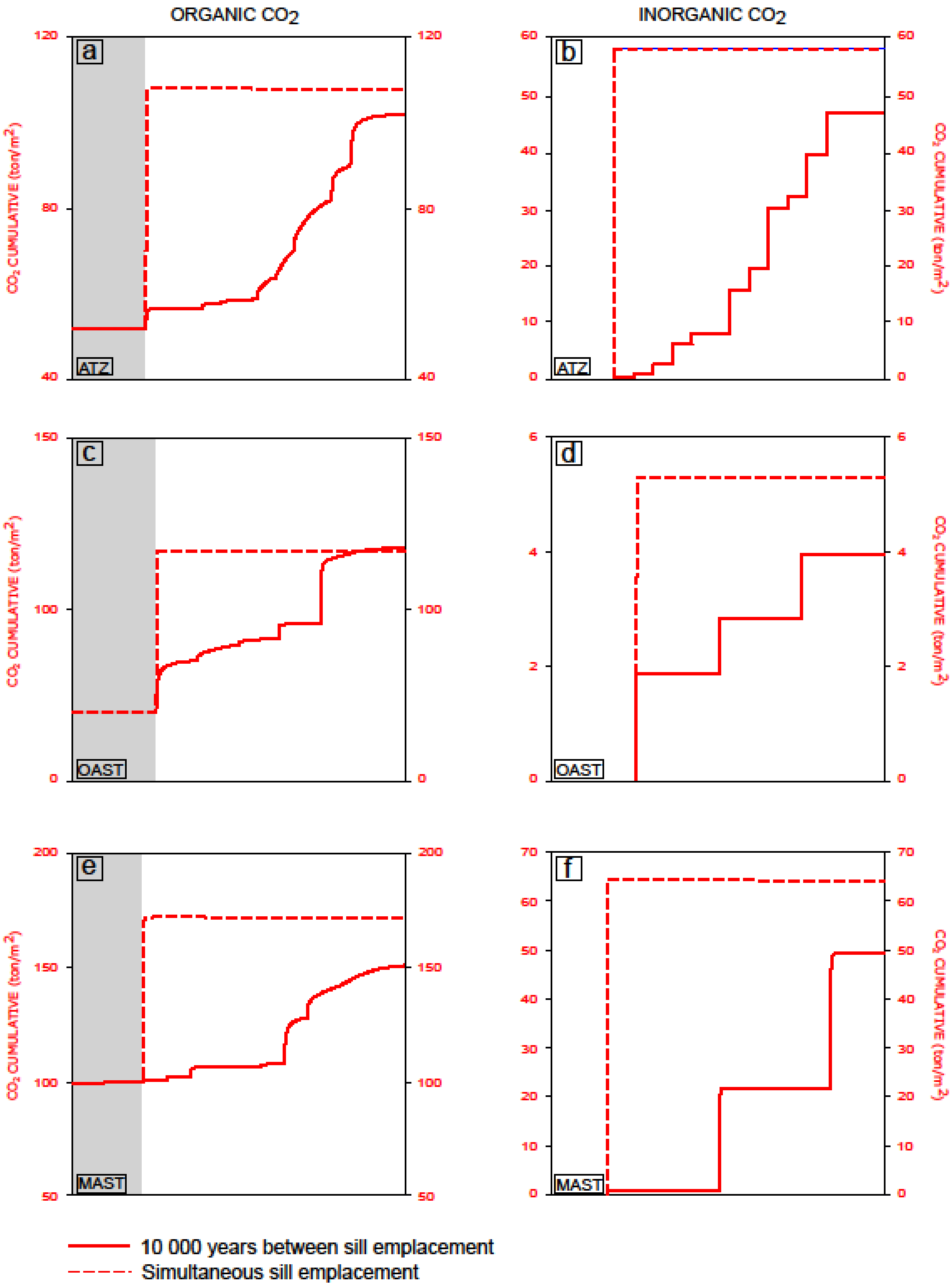

Modeled cumulative CO_2 production for simultaneous sill emplacement vs. sill emplacement every 10,000 years based on data from borehole ATZ (Fig. a; organic, Fig. b; inorganic), OAST (Fig. c; organic, Fig. d; inorganic) and MAST (Fig. e; organic, Fig. f; inorganic). Figures a, b, e and f show that for boreholes ATZ and MAST, the magnitude of generated CO_2 increases when selecting simultaneous sill emplacement in the model. The increase in CO_2 occurs because the sills are closely spaced in these boreholes. For borehole ATZ, the magnitude of organic and inorganic CO_2 after emplacement of the sills increase from 102 to 110 ton/m^2 (Fig. a) and from 47 to 58 ton/m^2 (Fig. b), respectively. For borehole MAST, organic and inorganic CO_2 increase from 154 to 172 ton/m^2 (Fig. e) and from 49 to 64 ton/m^2 (Fig. f), respectively. For borehole OAST, the organic and inorganic CO_2 production is more or less the same (note that the increase in inorganic CO_2 is only 1.3 ton/m^2; Fig. d).

Supplementary Table S1

Overview of studied boreholes and available sill thickness data.

Borehole	Basin	Log	Samples	Borehole depth	Sills	Vol. sills	Max. c.t.	Max. i.t.	Evaporite	Carbonate	Sills in U. Pal.	Sills in L. Pal.
				m	#	%	m	m	m	m	#	#
**ATZ**	AM	Yes	Yes	2622	12	18	482	88	507	220	12	0
**MAST**	AM	Yes	No	1868	9	20	367	131	3	167	5	4
**OAST**	AM	Yes	Yes	3097	5	8	255	143	375	109	3	2
**MA**	AM	Yes	Yes	3056	11	14	350	70	109	220	8	3
**IB**	AM	Yes	Yes	1350	2	11	91	70	0	0	0	2
**IBST**	AM	No	Yes	1358	1	NA	NA	NA	NA	NA	NA	NA
**SOL**	SO	No	Yes	NA	1	NA	NA	NA	NA	NA	NA	NA

Vol. sills = volume% sills
Max. c.t. = maximum cumulative sill thickness
Max. i.t. = maximum individual sill thickness
U. Pal. = upper Paleozoic sediments
L. Pal. = lower Paleozoic sediments
AM = Amazonas Basin
SO = Solimões Basin

Supplementary Table S2

U-Pb data for subsurface mafic sills from the Amazonas and Solimões sedimentary basins, northern Brazil.

	Composition					Ratios						Age (Ma)				
Sample Fractions†	Pb_c‡ (pg)	Pb*‡ / Pb_c	Th / U	^{206}Pb§ / ^{204}Pb	^{208}Pb# / ^{206}Pb	^{206}Pb†† / ^{238}U	err (2σ%)	^{207}Pb†† / ^{235}U	err (2σ%)	^{207}Pb†† / ^{206}Pb	err (2σ%)	^{206}Pb / ^{238}U	err (2σ)	^{207}Pb / ^{235}U	^{207}Pb / ^{206}Pb	corr. coef.
**Sample 1-MA-1-PA-2883.18 (Amazonas Basin) *201.477 ± 0.062/0.11/0.24 Ma***																
z2	0.2	32.7	1.30	1625.0	0.411	0.031763	(.10)	0.22042	(.88)	0.05035	(.84)	**201.57**	**0.20**	202.3	210	0.38
z6	0.1	262.8	1.29	12940.3	0.410	0.031760	(.05)	0.21988	(.16)	0.05023	(.14)	**201.55**	**0.10**	201.81	204.8	0.50
z3	0.1	20.7	0.90	1131.5	0.287	0.031750	(.13)	0.21927	(1.24)	0.05011	(1.19)	**201.49**	**0.25**	201.3	199	0.43
z5	0.2	48.2	1.26	2404.2	0.399	0.031737	(.06)	0.21940	(.52)	0.05016	(.50)	**201.41**	**0.13**	201.41	201	0.34
z4	0.2	59.0	1.21	2970.6	0.384	0.031731	(.07)	0.21976	(.47)	0.05025	(.44)	**201.37**	**0.13**	201.71	206	0.40
**Sample Amostra 8 (Solimões Basin) *201.470 ± 0.089/0.13/0.25 Ma***																
z3	0.1	139.3	0.81	7019.4	0.223	0.544852	(.19)	15.5065	(.22)	0.20651	(.08)	2803.7	4.4	2846.9	2877.	0.91
z7	0.1	84.8	0.49	4808.9	0.139	0.385479	(.12)	7.04317	(.19)	0.13258	(.12)	2101.8	2.1	2116.9	2131.	0.74
z8	0.2	31.2	0.17	1925.9	0.049	0.357195	(.28)	6.17546	(.40)	0.12545	(.26)	1968.9	4.8	2001.0	2034.	0.75
z2	0.1	56.0	0.33	3412.6	0.097	0.245494	(.15)	3.04259	(.33)	0.08993	(.27)	1415.2	1.9	1418.4	1423.	0.60
z9	0.1	63.0	0.85	3319.0	0.263	0.112039	(.08)	1.66359	(.23)	0.10774	(.20)	684.58	0.54	994.7	1760.	0.48
z6	0.2	12.7	1.24	650.7	0.392	0.035297	(.20)	0.24467	(2.08)	0.05030	(2.01)	223.61	0.43	222.2	208	0.41
z4	0.2	36.1	1.05	1889.8	0.334	0.031751	(.10)	0.22112	(.80)	0.05053	(.76)	**201.50**	**0.19**	202.8	218	0.45
z10	0.2	62.6	1.09	3241.8	0.344	0.031748	(.06)	0.21997	(.39)	0.05027	(.37)	**201.48**	**0.12**	201.88	206.6	0.37
z5	0.4	32.8	1.05	1723.6	0.332	0.031739	(.10)	0.21969	(.80)	0.05022	(.77)	**201.42**	**0.19**	201.7	204	0.40

Notes: Corr. coef. = correlation coefficient. Age calculations are based on the decay constants of Jaffey et al. (1971).

† All analyses are single zircon grains and pre-treated by the thermal annealing and acid leaching (CA-TIMS) technique. Data used in age calculations are in bold.

‡ Pb_c is total common Pb in analysis. Pb* is radiogenic Pb concentration.

§ Measured ratio corrected for spike and fractionation only.

[#] Radiogenic Pb ratio.

[††] Corrected for fractionation, spike, blank, and initial Th/U disequilibrium in magma. Mass fractionation correction of 0.25%/amu ± 0.04%/amu (atomic mass unit) was applied to single-collector Daly analyses. All common Pb is assumed to be blank. Total procedural blank was less than 0.1pg for U. Blank isotopic composition: $^{206}Pb/^{204}Pb$ = 18.15 ± 0.47, $^{207}Pb/^{204}Pb$ =15.30 ± 0.30, $^{208}Pb/^{204}Pb$ = 37.11 ± 0.87.

Supplementary Table S3

Input data (sediments) from borehole ATZ for thermal modeling.

Formation Name	Lithology Type	Top Depth Meters	Age Ma	Density kg/m^3	Heat Cap. J/kg/C	Porosity Fraction	Thermal Cond. W/m/K	TOC Wt.%	Lat. Heat Org. kJ/kg	Erosion Time Ma	Thickness eroded Meters
**Solimões**	S, Sh	5	9	2462	850	0.15	1.7	0.1	376	3	10
**Alter do Chao**	S, Sh, Si	341	126	2462	850	0.15	1.7	0.1	376	11	1000
**Andirá**	S, Sh, Si	448	295	2462	850	0.05	1.7	0.5	376	200	900
**Nova Olinda**	S, E	610	300	2540	888	0.05	3.5	0.5	376	NE	NE
**Nova Olinda**	S, Sh, Si, E, C	648	301	2500	852	0.05	2.85	0.5	376	NE	NE
**Nova Olinda**	E	1060	304	2160	926	0.01	5.6	0	376	NE	NE
**Nova Olinda**	S, Sh, Si, E, C	1137	305	2500	852	0.05	2.85	0.5	376	NE	NE
**Nova Olinda**	E	1237	306	2160	926	0.01	5.6	0	376	NE	NE
**Nova Olinda**	S, Sh, Si, E, C	1256	307	2500	852	0.05	2.85	0.5	376	NE	NE
**Nova Olinda**	E	1280	308	2160	926	0.01	5.6	0	376	NE	NE
**Nova Olinda**	S, Sh, Si, E, C	1364	309	2500	852	0.05	2.85	0.5	376	NE	NE
**Nova Olinda**	E	1505	310	2160	926	0.01	5.6	0	376	NE	NE
**Nova Olinda**	S, Sh, Si, E, C	1528	311	2500	852	0.05	2.85	0.5	376	NE	NE
**Nova Olinda**	E	1555	312	2160	926	0.01	5.6	0	376	NE	NE
**Nova Olinda**	S, Sh, Si, E, C	1618	313	2500	852	0.05	2.85	0.5	376	NE	NE
**Itaituba**	S, Sh, Si, E, C	1833	315	2500	852	0.02	2.85	0.6	376	NE	NE

Heat Cap. = heat capacity
Thermal Cond. = thermal conductivity
TOC = total organic carbon
Lat. Heat. Org. = latent heat of organic material
S = sandstone, M = mudstone, Sh = shale, Si = siltstone, E =evaporite, C = carbonate
NE = not eroded

Supplementary Table S3 continued

Input data (sediments) from borehole ATZ for thermal modeling.

Formation Name	Lithology Type	Top Depth Meters	Age Ma	Density kg/m^3	Heat Cap. J/kg/C	Porosity Fraction	Thermal Cond. W/m/K	TOC Wt.%	Lat. Heat Org. kJ/kg	Erosion Time Ma	Thickness eroded Meters
**Monte Alegre**	S, Sh	2269	320	2462	827	0.05	1.7	0.5	376	NE	NE
**Faro**	S, Sh, Si	2295	330	2462	850	0.05	1.7	1.5	376	321	400
**Oriximiná**	S, Sh	2295	350	2462	850	0.05	1.7	1	376	NE	NE
**Curiri**	S, Sh	2337	360	2462	850	0.05	1.7	0.8	376	NE	NE
**Barreirinha 1**	Sh	2453	370	2375	850	0.05	1.7	1.5	376	NE	NE
**Barreirinha 2**	Sh	2500	375	2375	850	0.05	1.7	8	376	NE	NE
**Ererê**	S, Sh, Si	2542	380	2462	850	0.05	1.7	1.5	376	NE	NE
**Maecuru**	S, Sh, Si	2615	390	2462	850	0.05	1.7	0.5	376	NE	NE
**Jatapu**	S, Sh, Si	2815	395	2462	850	0.05	1.7	0.5	376	NE	NE
**Manacapuru**	S, Sh, Si	2935	410	2462	850	0.05	1.7	0.5	376	NE	NE
**Pitinga**	S, Sh, Si	3135	430	2462	850	0.05	1.7	2.3	376	NE	NE
**Nhamundá**	S, Sh, Si	3384	440	2462	850	0.05	1.7	0.2	376	NE	NE
**Autás-Mirim**	S, Sh, Si	3724	450	2462	850	0.05	1.7	0.1	376	NE	NE

Heat Cap. = heat capacity
Thermal Cond. = thermal conductivity
TOC = total organic carbon
Lat. Heat. Org. = latent heat of organic material
S = sandstone, Sh = shale, Si = siltstone
NE = not eroded

Supplementary Table S3 continued

Input data (sills) from borehole ATZ for thermal modeling.

Sill<br>#	Emp. Temp.<br>°C)	Top Depth<br>Meters	Ages<br>Ma	Density<br>kg/m^3	Heat Capacity<br>J/kg/C	Thickness<br>Meters	Thermal Cond.<br>W/m/K	Solidus<br>°C	Liquidus<br>°C	Lat. Heat Cryst.<br>kJ/kg
**1**	1100	555	201.5	2550a/2700b	850a/820b	55	2.1	950	1150	376
**2**	1100	643	201.49	2550a/2700b	850a/820b	5	2.1	950	1150	376
**3**	1100	653	201.48	2550a/2700b	850a/820b	18	2.1	950	1150	376
**4**	1100	685	201.47	2550a/2700b	850a/820b	36	2.1	950	1150	376
**5**	1100	735	201.46	2550a/2700b	850a/820b	25	2.1	950	1150	376
**6**	1100	771	201.45	2550a/2700b	850a/820b	4	2.1	950	1150	376
**7**	1100	780	201.44	2550a/2700b	850a/820b	88	2.1	950	1150	376
**8**	1100	885	201.43	2550a/2700b	850a/820b	82	2.1	950	1150	376
**9**	1100	1304	201.42	2550a/2700b	850a/820b	60	2.1	950	1150	376
**10**	1100	1603	201.41	2550a/2700b	850a/820b	15	2.1	950	1150	376
**11**	1100	2105	201.4	2550a/2700b	850a/820b	32	2.1	950	1150	376
**12**	1100	2190	201.39	2550a/2700b	850a/820b	65	2.1	950	1150	376

Emp. Temp. = emplacement temperature
Thermal Cond. = thermal conductivity
Lat. Heat. Cryst. = latent heat of crystallization
a = melt, b = solid

Supplementary Table S4

Input data (sediments) from borehole OAST for thermal modeling.

Formation Name	Lithology Type	Top Depth Meters	Age Ma	Density kg/m^3	Heat Cap. J/kg/C	Porosity Fraction	Thermal Cond. W/m/K	TOC Wt.%	Lat. Heat Org. kJ/kg	Erosion Time Ma	Thickness Eroded Meters
**Alter Do Chão**	S, Sh, Si	4	126	2462	850	0.15	1.7	0.1	376	11	1000
**Andirá**	S, Sh, Si	200	294	2462	850	0.05	1.7	0.5	376	200	1000
**Nova Olinda 1**	S, Sh, Si, E	200	305	2485	875	0.02	2.85	0.5	376	296	330
**Nova Olinda 2**	S, Sh, Si, E, C	720	308	2500	852	0.02	2.85	0.5	376	NE	NE
**Itaituba**	S, Sh, Si, E, C	1165	315	2500	852	0.05	2.85	0.6	376	NE	NE
**Monte Alegre**	S, Si	1460	320	2462	850	0.05	1.7	0.5	376	NE	NE
**Faro**	S, Sh	1489	330	2462	850	0.05	1.7	1.5	376	321	270
**Oriximiná**	S, Sh, Si	1618	350	2462	850	0.05	1.7	1	376	NE	NE
**Curiri**	Sh	1805	360	2375	850	0.05	1.7	0.8	376	NE	NE
**Barreirinha 1**	Sh	2010	370	2375	850	0.05	1.7	1.5	376	NE	NE
**Barreirinha 2**	Sh	2120	375	2375	850	0.05	1.7	8	376	NE	NE
**Ererê**	S, Sh, Si	2207	380	2462	850	0.05	1.7	1.5	376	NE	NE
**Maecuru**	S, Sh, Si	2317	390	2462	850	0.05	1.7	0.5	376	NE	NE
**Jatapu**	S, Sh, Si	2525	395	2462	850	0.05	1.7	0.5	376	392	120
**Manacapuru**	S, Sh, Si	2525	410	2462	850	0.05	1.7	0.5	376	400	10
**Pitinga**	S, Sh, Si	2725	430	2462	850	0.05	1.7	2.3	376	NE	NE
**Nhamundá**	S, Sh, Si	3036	440	2462	850	0.05	1.7	0.2	376	NE	NE
**Autás-Mirim**	S, Sh, Si	3336	450	2462	850	0.05	1.7	0.1	376	NE	NE

Heat Cap. = heat capacity
Thermal Cond. = thermal conductivity
TOC = total organic carbon
Lat. Heat. Org. = latent heat of organic material
S = sandstone, Sh = shale, Si = siltstone, E =evaporite, C = carbonate
NE = not eroded

Supplementary Table S4 continued

Input data (sills) from borehole OAST for thermal modeling.

Sill<br>#	Emp. Temp.<br>°C	Top Depth<br>Meters	Age<br>Ma	Density<br>kg/m^3	Heat Capacity<br>J/kg/C	Thickness<br>Meters	Thermal Cond.<br>W/m/K	Solidus<br>°C	Liquidus<br>°C	Lat. Heat Cryst.<br>kJ/kg
**1**	1100	260	201.50	2550a/2700b	850a/820b	143	2.1	950	1150	376
**2**	1100	533	201.49	2550a/2700b	850a/820b	48	2.1	950	1150	376
**3**	1100	1408	201.48	2550a/2700b	850a/820b	4	2.1	950	1150	376
**4**	1100	2177	201.47	2550a/2700b	850a/820b	3	2.1	950	1150	376
**5**	1100	2784	201.46	2550a/2700b	850a/820b	62	2.1	950	1150	376

Emp. Temp. = emplacement temperature
Thermal Cond. = thermal conductivity
Lat. Heat. Cryst. = latent heat of crystallization
a = melt, b = solid

Supplementary Table S5

Input data (sediments) from borehole MAST for thermal modeling.

Formation Name	Lithology Type	Top Depth Meters	Age Ma	Density kg/m^3	Heat Cap. J/kg/C	Porosity Fraction	Thermal Cond. W/m/K	TOC Wt.%	Lat. Heat of Org. kJ/kg	Erosion Time	Thickness eroded Meters
**Alter Do Chão**	S, Sh	3	126	2462	850	0.15	1.7	0.1	376	11	830
**Andirá**	S, Sh, Si	417	295	2462	850	0.05	1.7	0.5	376	200	1000
**Nova Olinda**	S, Sh, Si, C	417	305	2490	826	0.05	2.1	0.5	376	298	850
**Itaituba**	S, Sh, Si, C	560	315	2490	826	0.05	2.1	0.6	376	NE	NE
**Monte Alegre**	S, Sh	1333	320	2462	850	0.05	1.7	0.5	376	NE	NE
**Monte Alegre**	S, Sh, C	1365	325	2490	850	0.05	2.1	0.5	376	NE	NE
**Faro**	S, Sh, Si	1427	330	2462	850	0.05	1.7	1.5	376	321	200
**Oriximiniá**	S, Sh, Si	1615	350	2462	850	0.05	1.7	1	376	NE	NE
**Curiri**	S, Sh	2032	360	2462	850	0.05	1.7	0.8	376	NE	NE
**Barreirinha 1**	Sh	2245	370	2375	850	0.05	1.7	1.5	376	NE	NE
**Barreirinha 2**	Sh	2426	375	2375	850	0.05	1.7	8	376	NE	NE
**Ererê**	S, Sh, Si	2607	380	2462	850	0.05	1.7	1.5	376	NE	NE
**Maecuru**	S, Sh, Si	2857	390	2462	850	0.05	1.7	0.5	376	NE	NE
**Jatapu**	S, Sh, Si	3065	395	2462	850	0.05	1.7	0.5	376	NE	NE
**Manacapuru**	S, Sh, Si	3185	410	2462	850	0.05	1.7	0.5	376	NE	NE
**Pitinga**	S, Sh, Si	3385	430	2462	850	0.05	1.7	2.3	376	NE	NE
**Nhamundá**	S, Sh, Si	3634	440	2462	850	0.05	1.7	0.2	376	NE	NE
**Autás-Mirim**	S, Sh, Si	3974	450	2462	850	0.05	1.7	0.1	376	NE	NE

Heat Cap. = heat capacity
Thermal Cond. = thermal conductivity
TOC = total organic carbon
Lat. Heat. Org. = latent heat of organic material
S = sandstone, Sh = shale, Si = siltstone
NE = not eroded

Supplementary Table S5 continued

Input data (sills) from borehole MAST for thermal modeling.

Sill #	Emp. Temp. °C	Top Depth Meters	Age Ma	Density kg/m^3	Heat Capacity J/kg/C	Thickness Meters	Thermal Cond. W/m/K	Solidus °C	Liquidus °C	Lat. Heat Cryst. kJ/kg
**1**	1100	602	201.47	2550a/2700b	850a/820b	43	2.1	950	1150	376
**2**	1100	654	201.48	2550a/2700b	850a/820b	112	2.1	950	1150	376
**3**	1100	1024	201.49	2550a/2700b	850a/820b	131	2.1	950	1150	376
**4**	1100	1325	201.50	2550a/2700b	850a/820b	8	2.1	950	1150	376
**5**	1100	1350	201.51	2550a/2700b	850a/820b	3	2.1	950	1150	376
**6**	1100	1749	201.52	2550a/2700b	850a/820b	10	2.1	950	1150	376
**7**	1100	1765	201.53	2550a/2700b	850a/820b	40	2.1	950	1150	376
**8**	1100	1809	201.54	2550a/2700b	850a/820b	10	2.1	950	1150	376
**9**	1100	1851	201.55	2550a/2700b	850a/820b	10	2.1	950	1150	376

Emp. Temp. = emplacement temperature
Thermal Cond. = thermal conductivity
Lat. Heat. Cryst. = latent heat of crystallization
a = melt, b = solid

Supplementary Table S6

Present day total organic carbon (TOC) and vitrinite reflectance data from borehole OAST for thermal modeling.

Depth Meters	Formation Name	TOC Wt.%	Vitrinite %Ro	Depth Meters	Formation Name	TOC Wt.%	Vitrinite %Ro
1430	Itaituba	0.98	NA	2156	Barreirinha	5.52	NA
1514	Faro	NA	0.67	2189	Barreirinha	4.96	NA
1719	Oriximiná	NA	0.61	2558	Manacapuru	NA	0.81
1722	Oriximiná	1.63	NA	2559	Manacapuru	1.34	NA
1998	Cuiri	1.00	NA	2852	Pitinga	1.51	NA
2013	Barreirinha	0.80	NA	2883	Pitinga	1.61	NA
2033	Barreirinha	1.05	NA	2897	Pitinga	1.43	NA
2052	Barreirinha	1.10	NA	2930	Pitinga	1.43	NA
2070	Barreirinha	0.95	NA	2951	Pitinga	1.16	NA
2082	Barreirinha	NA	0.75	2979	Pitinga	1.04	NA
2088	Barreirinha	1.17	NA	2997	Pitinga	1.48	NA
2105	Barreirinha	1.73	NA	3011	Pitinga	1.12	NA
2121	Barreirinha	3.64	NA	3033	Pitinga	1.08	NA
2141	Barreirinha	4.95	NA				

NA = not available

Supplementary Table S7

Organic and inorganic CO_2 fluxes per sill emplacement from thermal modeling.

Sill #	Age Ma	Formation Name	Top Depth sill Meters	Thickness sill Meters	Flux organic kg/m^2 per year	Flux inorganic kg/m^2 per year
**ATZ**						
**1**	201.50	Andira	555	55	328	52
**2**	201.49	Nova Olinda	643	5	15	442
**3**	201.48	Nova Olinda	653	18	47	1190
**4**	201.47	Nova Olinda	685	36	82	1584
**5**	201.46	Nova Olinda	735	25	8	393
**6**	201.45	Nova Olinda	771	4	4	72
**7**	201.44	Nova Olinda	780	88	39	1259
**8**	201.43	Nova Olinda	885	82	2	350
**9**	201.42	Nova Olinda	1304	60	118	1327
**10**	201.41	Nova Olinda	1603	15	101	1238
**11**	201.40	Itaituba	2105	32	363	1936
**12**	201.39	Itaituba	2190	65	93	1911

Supplementary Table S7 continued

Organic and inorganic CO_2 fluxes per sill emplacement from thermal modeling.

Sill<br>#	Age<br>Ma	Formation<br>Name	Top Depth sill<br>Meters	Thickness sill<br>Meters	Flux organic<br>kg/m^2 per year	Flux inorganic<br>kg/m^2 per year
**MAST**						
**1**	201.55	Oriximiná	1851	10	545	0
**2**	201.54	Oriximiná	1809	10	573	0
**3**	201.53	Oriximiná	1765	40	64	0
**4**	201.52	Oriximiná	1749	10	0	0
**5**	201.51	Monte Alegre	1350	3	182	0
**6**	201.50	Itaituba	1325	8	325	711
**7**	201.49	Itaituba	1024	131	402	1203
**8**	201.48	Itaituba	654	112	285	2235
**9**	201.47	Itaituba	602	43	0	0
**OAST**						
**1**	201.50	Nova Olinda	260	143	356	190
**2**	201.49	Nova Olinda	533	48	95	149
**3**	201.48	Itaituba	1408	4	315	1122
**4**	201.47	Barreirinha	2177	3	2794	0
**5**	201.46	Pitinga	2784	62	1080	0

Supplementary text

Input data for thermal sill model

The lithological layers described in Supplementary Tables S3-S5 (e.g. Alter do Chão) correspond to already established sedimentary formations present within the Amazonas Basin[1,2]. The Amazonas Basin comprise up to 17 sedimentary formations, including (from stratigraphically deepest to shallowest) Autás-Mirim, Nhamundá, Pitinga, Manacapuru, Jatapu, Maecuru, Ererê, Barreirinha, Cuiri, Oriximiná, Faro, Monte Alegre, Itaituba, Nova Olinda, Andirá, Alter do Chão and Solimões. The boreholes in which the thermal modeling is based on do not necessarily include all these formations, either because the layers are eroded or because the boreholes are not deep enough, and failed to recover the deepest formations. In order to account for the full depositional history of the basin, these layers have also been added (Supplementary Tables S3-S5; red color). The thicknesses of the missing formations and thicknesses of eroded sediments of formations that are present, were estimated based on previously published data from boreholes from the Amazonas Basin[2]. The Solimões Formation was only deposited in the western part of the Amazonas Basin[7], thus this layer is not added to boreholes MAST and OAST, which are located toward the eastern part of the basin. The timing of erosion is from ref. 7. As seen in the Supplementary Tables S3-S5, the different formations generally comprise more than one lithology. Properties such as thermal conductivity, density and heat capacity vary for different sediment types. Ideally, each rock type should correspond to one individual lithological layer in the model, however the rock types are interlayered

with quite high frequency within the formations, making this difficult. In order for the input data to be representative for each formation, values for thermal conductivity, density and heat capacity[3,4] represent an average value of the different rock types present in each formation. TOC and vitrinite reflectance data in Supplementary Tables S3-S6 were provided by the National Petroleum Agency in Brazil. Input data for latent heat of organic maturation and input data for the sills are from ref. 6.

Supplementary references

[1.] Milani, E. J., & Zalan, P. V. An outline of the geology and petroleum systems of the Paleozoic interior basins of South America. *Episodes*, *22*, 199-205 (1999).

[2.] Cunha, P. R. C., Melo, J. H. G, & da Silva, O. B. Bacia do Amazonas. *Boletim de Geociências da PETROBRAS*, *15*, 227-251 (2007).

[3.] Aarnes, I., Fristad, K., Planke, S., & Svensen, H. The impact of host-rock composition on devolatilization of sedimentary rocks during contact metamorphism around mafic sheet intrusions. *Geochemistry, Geophysics, Geosystems*, *12*(10) (2011).

[4] Clauser, C., & Huenges, E. Thermal conductivity of rocks and minerals. *Rock physics & phase relations: a handbook of physical constants*, 105-126 (1995).

[5.] Caputo, M. V., & Suares, E. A. A. Eustatic and tectonic change effects in the reversion of the transcontinental Amazon River drainage system. *Brazilian Journal of Geology*, *46*(2), 301-328 (2016).

[6.] Svensen, H. H., Iyer, K., Schmid, D. W., & Mazzini, A. Modelling of gas generation following emplacement of an igneous sill below LUSI, east Java, Indonesia. *Marine and Petroleum Geology*, in press.
